# Evidence for Light and Tissue Specific Regulation of Genes Involved in Fructan Metabolism in *Agave tequilana*

**DOI:** 10.3390/plants11162153

**Published:** 2022-08-19

**Authors:** Alan D. Gomez-Vargas, Karen M. Hernández-Martínez, Macrina E. López-Rosas, Gerardo Alejo Jacuinde, June Simpson

**Affiliations:** Department of Genetic Engineering, CINVESTAV Irapuato, Km. 9.6 Libramiento Norte Carretera Irapuato-León, Irapuato 36821, Guanajuato, Mexico

**Keywords:** *A. tequilana*, fructan metabolism, gene structure, promoter motifs, light induction, transcription factors

## Abstract

Plant Glycoside Hydrolase Family 32 (PGHF32) contains the fructosyltransferases and fructan exohydrolase enzymes responsible for fructan metabolism, in addition to closely related vacuolar and cell wall acid invertases. *Agave* species produce complex and dynamic fructan molecules (agavins) requiring 4 different fructosyltransferase activities (1-SST, 1-FFT, 6G-FFT and 6-SFT) for their synthesis. Combined analysis of RNAseq and genome data for *A. tequilana* led to the characterization of the genes encoding 3 fructosyltransferases for this species and support the hypothesis that no separate 6-SFT type enzyme exists in *A. tequilana,* suggesting that at least one of the fructosyltransferases identified may have multiple enzymatic activities. Structures for PGHF32 genes varied for *A. tequilana* and between other plant species but were conserved for different enzyme types within a species. The observed patterns are consistent with the formation of distinct gene structures by intron loss. Promoter analysis of the PGHF32 genes identified abundant putative regulatory motifs for light regulation and tissue-specific expression, and these regulatory mechanisms were confirmed experimentally for leaf tissue. Motifs for phytohormone response, carbohydrate metabolism and dehydration responses were also uncovered. Based on the regulatory motifs, full-length cDNAs for MYB, GATA, DOF and GBF transcription factors were identified and their phylogenetic distribution determined by comparison with other plant species. In silico expression analysis for the selected transcription factors revealed both tissue-specific and developmental patterns of expression, allowing candidates to be identified for detailed analysis of the regulation of fructan metabolism in *A. tequilana* at the molecular level.

## 1. Introduction

Fructans, synthesized by approximately 15% of angiosperms, are a structurally diverse group of molecules made up of multiple fructose units linked to a precursor sucrose molecule [1,2,3]. The main biological function of fructans is considered to be as a reserve carbohydrate, although it has also been suggested that fructan metabolism in plants arose as an evolutionary adaptation to inhabit environments with prolonged periods of drought or low water availability [1,4,5,6,7]. More recently, the roles of fructans in osmoregulation and signaling have also been described [8,9,10,11].

The *Agave* genus comprises plants with the ability to accumulate high levels of fructans and these carbohydrates are exploited commercially for the production of tequila, mezcal, pulque and syrups [12,13,14]. Agave fructans or agavins, are the most complex of all fructans in terms of their highly branched structures [15,16], and their synthesis by fructosyltransferases (FT) requires 4 different enzymatic activities: sucrose: sucrose 1-fructosyltransferase (1-SST), fructan: fructan 1-fructosyltransferase (1-FFT), sucrose: fructan 6 -fructosyltransferase (6-SFT) and fructan: fructan 6G-fructosyltransferase (6G-FFT). In contrast, fructan exohydrolase enzymes (FEH) are responsible for fructan degradation [17]. Plant FTs and FEHs belong to Plant Glycoside Hydrolase Family 32 (PGHF32), which also includes vacuolar and cell wall invertases [2,18,19]. Enzymes belonging to this family are characterized by highly similar and conserved amino acid sequences. Phylogenetic analyses of these enzymes have suggested that FTs evolved from vacuolar invertases, while FEHs are more closely related to cell wall invertases [18,19].

In *Agave tequilana* Weber var. Azul, extensive transcriptome and gene expression analysis has shown that PGHF32 genes show specific and differential patterns of expression during flowering and in distinct plant tissues [20,21,22]. In addition, analysis of in vitro grown seedlings revealed that 1-SST and 1-FFT transcripts accumulate in response to the exogenous application of sucrose, abscisic acid (ABA) and other plant hormones (salicylic acid, kinetin and methyl jasmonate) associated with responses to abiotic stress [23,24]. Likewise, it has been shown that fructan synthesis in plants such as wheat and chicory is regulated by metabolic, developmental and environmental stimuli, including light, drought, sucrose and abscisic acid [25,26,27,28,29,30]. These reports of the diverse stimuli that modify the expression of PGHF32 genes imply complex interactions between individual genes and an array of different transcription factors where potential candidates are MYB, associated with drought responses; GATA and GBF, associated with light responses; and DOF, associated with carbon metabolism, among many others. At the molecular level, however, data is only available for transcription factor (TF) R2R3 type MYB genes studied in wheat (*Triticum aestivum)* and chicory (*Chicorium intybus*). In wheat TaMYB13-1, TaMYB13-2 and TaMYB13-3 transcription factors interact with and induce the expression of the wheat Ta1-SST, Ta6-SFT and Ta1-FFT genes in stem tissue and are induced by sucrose [25]. In chicory, CiMYB-3 and CiMYB-5 induce the expression of the Ci1-FEH1, Ci1-FEH2a, and Ci1-FEH2b genes encoding enzymes for fructan degradation without affecting the expression of genes encoding fructan synthetic enzymes, while CiMYB-17 was identified to be capable of regulating genes for both fructan synthesis and degradation under abiotic stress conditions (drought and freezing) [25,31].

*Agave* species are some of the few plant species that combine both fructan metabolism and Crassulacean Acid Metabolism (CAM) [14,32]. It is therefore of interest to understand the factors that regulate fructan metabolism in these species and how fructan metabolism is integrated into CAM and general carbohydrate metabolism. Fructan metabolism is strongly linked with sucrose metabolism, since sucrose is crucial for fructan synthesis through PGHF32 enzymes [3]. The tissue-specific expression patterns under biotic and abiotic stimuli suggest that the genes that encode the PGHF32 enzymes involve a complex regulation directed by multiple transcription factors; therefore, determination of the gene structures and putative regulatory motifs in the promoter regions of the members of PGHF32 in *A. tequilana* would allow us to address the evolution of PGHF32 and in particular the enzymes associated with the metabolism of fructans. The identification and characterization of genes encoding transcription factors putatively involved in the regulation of PGHF32 in *A. tequilana* is one of the first steps to understand in detail the regulation of fructan metabolism at the molecular level and to apply this knowledge to future biotechnological applications.

Recently, a draft genome for *A. tequilana* became available (Herrera-Estrella et al. in preparation) and based on this data, we determined the gene structure and identified putative regulatory promoter motifs for each of the members of PGHF32 from *A. tequilana.* Here, we report the results of these analyses and also the in silico identification and characterization of several *A. tequilana* transcription factor cDNAs and predicted proteins. We also confirm light-inducible and tissue-specific regulation of selected PGHF32 encoding genes.

## 2. Methods

### 2.1. Characterization of Genes Encoding PGHF32 Members in Agave tequilana

*Agave tequilana* genomic sequences were obtained using the BLAST algorithm [33] in the *A. tequilana* draft genome (Herrera-Estrella et al. unpublished), permission to access the data prior to publication can be arranged by directly contacting Dr. Alfredo Herrera-Estrella: (alfredo.herrera@cinvestav.mx) or Dr. Selene Fernández Valverde: (selene.fernandez@cinvestav.mx) by using as queries the cDNA sequences of PGHF32 previously reported. Genome coordinates were determined for each cDNA to obtain the genomic sequence. An alignment of the genomic and cDNA sequences was carried out using the MUSCLE algorithm [34] in SeaView software version 5.0.2.Available online: http://doua.prabi.fr/software/seaview (accessed on 24 May 2022) in order to determine the exon/intron structure for each genomic sequence. These analyses were also carried out for members of PGHF32 from a selection of fructan and non-fructan accumulating species: asparagus (*Asparagus officinalis)*, goatgrass *(Aegilops tauschii*)*,* wheat (*Triticum aestivum)*, barley (*Hordeum vulgare)*, *Arabidopsis thaliana*, carrot (*Daucus carota*), beetroot (*Beta vulgaris*), maize (*Zea mays*) and rice (*Oryza sativa*). Genomic sequences were obtained from “Ensembl Plants”. Available online: https://plants.ensembl.org/index.html (accessed on 22 August 2020) and “The Arabidopsis Information Resource” (TAIR). Available online: https://www.arabidopsis.org/data-bases (accessed on 28 August 2020). Enzymes and accession numbers are listed in Supplementary Table S1.

### 2.2. Identification of Regulatory Motifs in the Promoters of PGHF32

Based on the coordinates determined for the PGHF32 genes, a 2 kb region upstream to the transcription start site was analyzed using the PLACE “Plant Cis-Regulatory DNA Elements” database [35]. Available online: https://www.dna.affrc.go.jp/PLACE/?action=newplace (accessed on 6 November 2021) that identifies putative motifs associated with characterized transcription factors. Identified motifs were separated into groups based on regulatory function such as light regulation, response to phytohormones, etc. Detailed comparisons were then carried out for selected motifs related to light regulation, tissue-specific regulation, phytohormone responses, regulation of carbohydrate metabolism and responses to dehydration.

### 2.3. A. tequilana Transcriptome Database Searches

A search was carried out using the BLAST algorithm in the *A. tequilana* transcriptome reported by [21], using experimentally validated transcription factors as queries for MYB, GATA, DOF and GBF type transcription factors. The best hits with e < 10^−5^ were chosen and the open reading frames (ORF) of the transcripts were determined by using ORF finder. Available online: http://www.bioinformatics.org/sms2/orf_find.html (accessed on 22 June 2021). Sequences encoding the complete predicted proteins were selected as novel transcription factor encoding sequences from *A. tequilana* and were used for comparison with previously characterized amino acid sequences of the same transcription factors from other species. Accession numbers for sequences used in these analyses are listed in Supplementary Table S2 and newly identified *A. tequilana* sequences were deposited in the GenBank database.

### 2.4. Alignment and Identification of Conserved Motifs

Complete predicted protein sequences were selected for alignment using the MUSCLE algorithm and conserved motifs were compared for each member of the transcription factor family analyzed. Alignments and phylogenetic analyses for MYB, GATA, DOF and GBF transcription factors were performed using Geneious^®^ (version 6.1.8). Available online: www.geneious.com (accessed on 15 February 2022), taking into account all sites including gaps/missing data with the UPGMA algorithm and the Jukes-Cantor genetic distance model with a bootstrap analysis using a total of 1000 repetitions and a support threshold of 50%. PGHF32 phylogenetic analysis was carried out using all sites of the amino acid sequences, including gaps/missing data with the Maximum likelihood algorithm by using the Molecular Evolutionary Genetics Analysis (MEGA 11) software [36]. According to amino acid information, the best substitution model was obtained (WAG + G + I) and used to construct the dendrograms with a bootstrap analysis using a total of 1000 repetitions.

### 2.5. In Silico Expression Analysis

Expression levels of transcripts from each individual transcription factor in different tissues were determined in silico and expressed as “transcripts per million” (TPM), as described by [20,21]. Heatmaps were created based on transcription factor expression data from identified *A. tequilana* transcripts using the ggplot2 function from the tidyverse library in the RStudio statistics package version 1.4.1717. Available online: https://www.rstudio.com/products/rstudio/download/ (accessed on 8 May 2022).

### 2.6. qRT-PCR Analysis

Total RNA was isolated from green leaf sections of 3 individual 3-year-old *A. tequilana* plants using the TRIzol reagent (Invitrogen; Carlsbad, CA, USA) and the PureLink RNA mini kit (Invitrogen) according to the manufacturer’s protocol. For light/dark regimes, green leaf tissue was collected at time 0 before subjecting 2 of the plants to complete darkness for 7 days. A control plant was left under normal light cycle conditions (16 h light/8 h darkness). After 7 days, RNA was again extracted from green leaf tissue and the plants were returned to a normal light cycle for 48 h, after which green leaf RNA was again extracted. For tissue-specific analysis, RNA was extracted from green and white sections of the same leaves (Supplementary Figure S1). qRT-PCR analysis was carried out on 5 members of *A. tequilana* PGHF32: *Atq1SST-1*, *Atq6G-FFT-1* and *Atq6G-FFT-2*, *AtqFEH-4* and *AtqVinv-1* using primers described in [30,37]. cDNA templates were synthesized by incubating 1 μg of total RNA with dT primer and revertaid reverse transcriptase (Thermo Scientific) according to the manufacturer’s instructions. qRT-PCR was carried out by using Kapa Sybr Fast qPCR master mix (2X) Universal reagent (Sigma Aldrich, St. Louis, MO, USA) in a StepOne Plus thermocycler (Applied Biosystems, Foster City, CA, USA). Thermal conditions and the relative quantification were determined as described by [22].

## 3. Results

### 3.1. Determination of Gene Structures for A. tequilana PGHF32 Encoding Genes

A previous report described the gene structures for 6 of the members of *A. tequilana* PGHF32 [37]. However, more recent RNAseq results revealed several new members of PGHF32 for which gene structures had not been determined [20,21], and in this analysis a further 3 genes encoding vacuolar invertases were identified by analysis of the draft genome (denominated *AtqVinv like 3, 4* and *5*). Genome analysis did not uncover genes encoding 6-SFT type enzymes, supporting the absence of 6-SFT type transcripts in the previous RNAseq analysis and suggesting that in *A. tequilana* (and perhaps more widely in the *Agave* genus), no separate enzymes with 6-SFT activity exist. However, given the complexity of agavin structures, this activity must therefore be carried out by the previously described 1-SST, 6G-FFT or 1-FFT enzymes.

**Figure 1:**
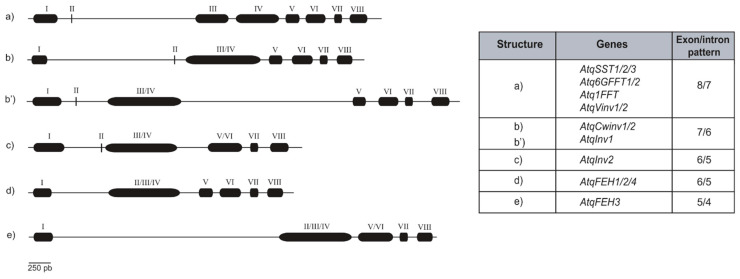
Schematic representation of the genomic structures of *A. tequila* PGHF32 encoding genes. Introns are represented by straight lines and exons with solid boxes. Roman numerals (I-VIII) from left to right identify each exon. Scale bar represents 250 base pairs. The table summarizes the genes and their exon/intron structure.

By comparison with transcriptome-derived cDNAs, the structures of each of the *A. tequilana* PGHF32 family genes were determined, confirming the structures previously described by Cortés Romero et al. [37]. Independent genome coordinates could not be determined for *AtqCwinv-3* and *AtqCwinv-2* previously identified from RNAseq data, suggesting that these isoforms actually represent alleles of a single locus. Six different patterns of exon/intron structure were determined, as shown in Figure 1. The 9-nucleotide mini-exon (Exon II), characteristic of PGHF32, was identified in all *A. tequilana* PGHF32 genes, with the exception of those encoding the *A. tequilana* FEH enzymes. The 3 amino acids encoded by Exon II comprise a highly conserved motif characteristic of PGHF32 with the consensus sequence DPN, where the aspartic acid residue (D) is essential for the catalytic activity of the PGHF32 enzymes. The nucleotide ranges of exons and introns are shown in Supplementary Table S3.

FT and Vinv genes are the only members of the family with 8 well-defined exons separated by 7 introns (Figure 1a), whereas the rest of the genes present fusions of some exons, specifically exons III/IV in *AtqCwinv1/2* and *AtqInv1* genes (Figure 1b) and exons II/III/IV in the *AtqFEH* genes (Figure 1d). Additionally, *AtqInv2* and *AtqFEH3* present a fusion of exons V/VI (Figure 1b,e). The changes in gene structure produced by the fusion of different exons are consistent with intron loss.

To determine whether the gene structures for PGHF32 were conserved across different species and taxonomic levels, a comparison with PGHF32 gene structures from monocotyledonous fructan producing species: *A. tequilana,* asparagus (*Asparagus officinalis)*, goatgrass *(Aegilops tauschii*)*,* wheat (*Triticum aestivum)*, barley (*Hordeum vulgare)* and monocotyledonous and dicotyledonous fructan non-producing species: *Arabidopsis thaliana*, carrot (*Daucus carota*), beetroot (*Beta vulgaris*), maize (*Zea mays*) and rice (*Oryza sativa*) was carried out. The dendrogram shows two main clades: A, containing FTs and vacuolar invertases and B, containing FEH and cell wall invertases. Clade A can further be classified into 4 subclades, all with bootstrap values between 97 and 100. Subclade A/a contains sequences from the gramineous monocotyledons, subclade A/b contains non-gramineous monocotyledons from the Asparagales order (*A. tequilana* and *A. officinalis*), subclade A/c gramineous and non-gramineous monocotyledons and subclade A/d contains dicotyledonous species. Within the subclades, defined clades are also observed that separate enzymes with fructosyltransferase activity from those with vacuolar invertase activity, for example, subclades A/a/1 and A/a/2. In clade B, 3 subclades can also be identified and were denoted as B/e, B/f and B/g. Subclade B/e is specific to dicotyledonous species and supported by a bootstrap value of 100. Subclade B/g is specific to monocotyledonous species, whereas subclade B/f contains both monocotyledonous and dicotyledonous species. However, the node leading to subclades f and g has a very low Boostrap of 52, suggesting that the topology of this section of the dendrogram is less robust. In contrast to clade A in clade B, no clear separation of clades containing enzymes with different activities or specific to gramineous and non-gramineous monocotyledons was observed and although subclade e is specific to dicotyledons, it contains enzymes with different functions (both Cwinv and FEH).

**Figure 2:**
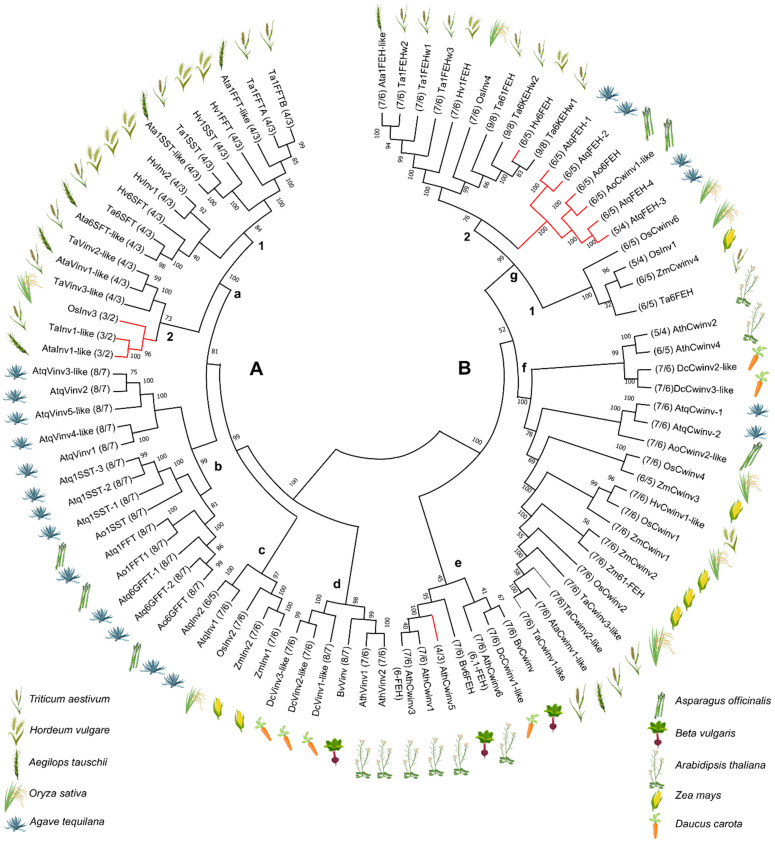
Dendrogram showing relationships between *A. tequilana* PGHF32 enzymes and those of other plant species. Upper case letters indicate the Vacuolar invertase/Fructosyltransferase clade (A) and the Cell wall invertase/FEH clade (B). Lower case letters indicate different subclades within clades A and B. Numbers within the dendrogram represent the Bootstrap analysis values and the numbers followed by the name represent the gene structure (exons/introns). Phylogenetic analysis was carried out by using amino acid sequences and considering all sites including gaps/missing information based on the Maximum likelihood method and the WAG+G+I substitution model. Genes where the mini exon is not present are indicated by red colored branches. Symbology represents the species analyzed.

As shown in Supplementary Table S4, the 7 exon/6 intron pattern is the only conserved pattern in all species. Other patterns identified consisted of 4/3, 3/2, 8/7, 6/5 and 9/8 exon/intron conformations, although no consistency was observed in exon/intron patterns regarding taxonomic classification, for example within the monocotyledons or dicotyledons or within the Poales order. However, *A. tequilana* and *A. officinalis*, both of the Asparagales order and non-gramineous monocotyledonous species, share the 8/7, 7/6 and 6/5 exon/intron pattern conformations. The mini-exon is also absent in the *A. officinalis* FEH gene, as observed for *A. tequilana*. Interestingly, when the exon/intron patterns are superimposed on a dendrogram showing the relationships between the genes listed in Supplementary Table S3, a good association between clades and exon/intron structures is observed even across different species, as indicated by the exon/intron patterns (4/3, 3/2, 8/7, 6/5, 7/6 and 9/8) in Figure 2.

Some changes in exon/intron structure are directly due to the fusion of the 9-nucleotide mini-exon with the previous or following exon. An example is subclade A/a containing vacuolar invertase and fructosyltransferase enzymes from *A. tauschii*, wheat, rice and barley. Most of the genes encoding these enzymes have a 4 exon/3 intron structure, whereas 3 members, marked in red, have a 3 exon/2 intron structure. This structural change is due to the fusion of the mini exon with the previous exon. Loss of a discrete mini exon was also observed for the *A. thaliana* Cwinv enzymes in clade B/e and for Cwinv and FEH type enzymes from *A. tequilana*, *A*. *officinalis* and *H. vulgare* in clade B/g/2 (Figure 2).

In general, the mini-exon was present in all types of PGHF32 members (fructosyltransferases, cell wall and vacuolar invertases and fructan exohydrolases with the exception of the *A. tequilana* and *A. officinalis* FEH genes. In *A. tequilana AtqInv2*, *A. officinalis Ao1-SST* and beetroot *Bv6FEH,* however, the mini-exon encoded 2 rather than 3 amino acids, where the first amino acid of the triad was encoded on the previous exon. Additionally, the third amino acid of the mini-exon triad was found to be variable among the enzymes, encoding either Asparagine (N), Cysteine (C) or Serine (S) (Supplementary Table S3).

### 3.2. Identification of Putative Regulatory Motifs in the Promotor Regions of Genes Encoding PGHF32 Enzymes in A. tequilana

**Figure 3:**
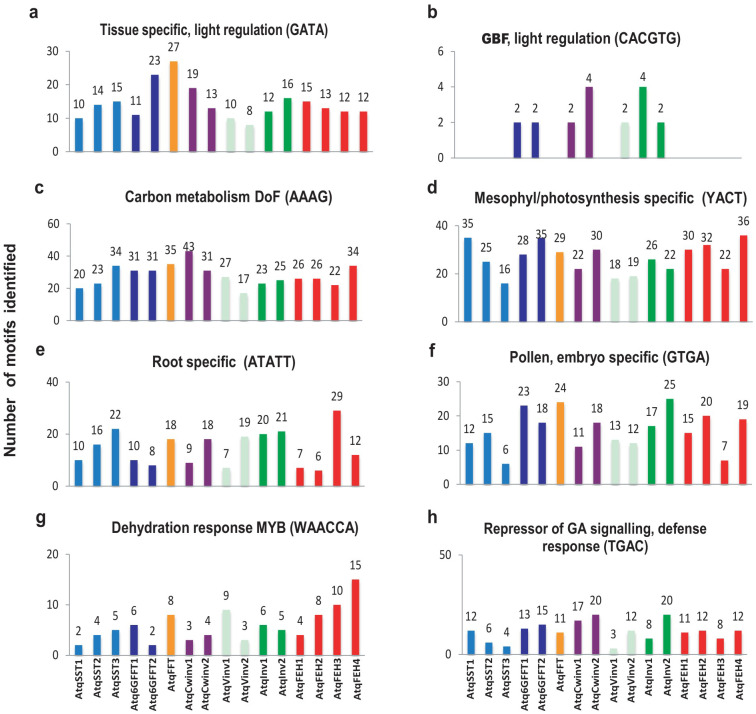
Abundance of transcription factor regulatory motifs identified in PGHF32 promoter regions. Conserved motifs and their associated regulatory process are indicated above each graph. PGHF32 members are indicated at the foot of each column on the x-axis and the y-axis shows the numbers of motifs identified. Each color indicates the different PGHF32 members for present in the *A. tequilana* genome.

To locate regulatory motifs denoting putative transcription factor binding sites, 2 Kb regions at the 5’ UTR upstream from the coding sequence of 16 *A. tequilana* PGHF32 genes were analyzed. The PLACE program identified 149 putative motifs across all 16 genes. To simplify the comparison across genes, motifs were placed in 24 different groups according to the regulatory process with which they are associated. The numbers of motifs identified for each group showed a similar pattern across all the genes and a typical example of these results is shown for the 4 *A. tequilana* FEH genes in Supplementary Figure S2. As can be seen, the most abundant group of motifs was associated with light regulation, followed by tissue-specific expression, pollen-specific expression, and expression related to phytohormones. In order to analyze motif patterns in more detail, the abundance of selected motifs was compared between the individual genes, as shown in Figure 3.

With the exception of the G-box binding factor (GBF) motif involved in light regulation, the chosen motifs were present in all the *A. tequilana* PGHF32 genes. Differences in abundance may be between the different types of genes; for example, the DOF and GATA motifs are more prevalent in the FT encoding genes than in the other PGHF32 genes. Differences are also observed within the same gene type; for example, the YACT motif is less abundant in *Atq1SST-3* in comparison to *Atq1SST-1* and *2* and the ATATT motif is more abundant in *AtqFEH-3* in comparison to the other FEH genes. Interestingly, although the GBF motif (CACTGT) is known to be necessary for light regulation in many plant species [38,39,40], only 2 or 4 copies of this element were found and only in *Atq6GFFT-1* and *2*, *AtqCwinv-1* and *2*, *AtqInv1* and *2* and *AtqVinv-2*. The MYB motif (WAACCA), also previously reported to be involved in the regulation of PGHF32 genes [26,28], was also less abundant in comparison to the other motifs.

**Figure 4:**
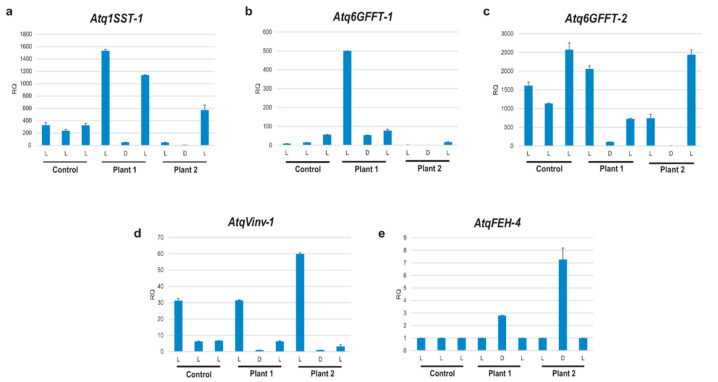
qRT-PCR analysis of PGHF32 genes during light and dark treatments. L, Normal light conditions (16 h light/8 h darkness), D, dark treatment for 7 days. Plants 1 and 2 were placed under normal light conditions (L), then under darkness (D) for 7 days and finally they were moved back to light conditions (L) for 48 h. Error bars indicate the standard deviation. The genes analyzed are indicated above each graph.

To confirm the predicted light- and tissue-specific regulation of *A. tequilana* PGHF32 genes, a simple expression analysis was carried out by qRT-PCR using leaf tissue from 3-year-old *A. tequilana* plants. As shown in Figure 4, plants subjected to 7 days in complete darkness showed a decrease in the expression levels of all fructosyltransferase genes (*Atq1SST-1, Atq6GFFT-1* and *2*) as did the vacuolar invertase gene *AtqVinv-1*. The fructan exohydrolase gene *AtqFEH-4* is expressed at low levels in the light but shows a slight induction under darkness. In contrast, when the plants were returned to normal light conditions for 48 h, expression of all genes increased, with the exception of *AtqFEH-4,* which was repressed, confirming a pattern of light-dependent transcriptional regulation. To address the question of tissue-specific patterns of expression, qRT-PCR analysis was carried out on green aerial leaf tissue and white tissue from the leaf base of the same leaf (Supplementary Figure S3). In this case, the fructosyltransferase genes (*Atq1SST-1, Atq6GFFT-1* and *2*) showed increased levels of expression by several thousand fold in white tissue as compared to green tissue and *AtqFEH-4* showed an increase in expression between 2 and 6 fold; however, the vacuolar invertase gene (*AtqVinv-1*) was repressed in white tissue. These results therefore also confirm that the *A. tequilana* PGHF32 genes are transcriptionally regulated in a tissue-specific manner in leaf tissue.

### 3.3. Identification and Analysis of Coding Sequences and Predicted Proteins for Transcription Factors Putatively Involved in the Regulation of PGHF32 Genes in A. tequilana

**Figure 5:**
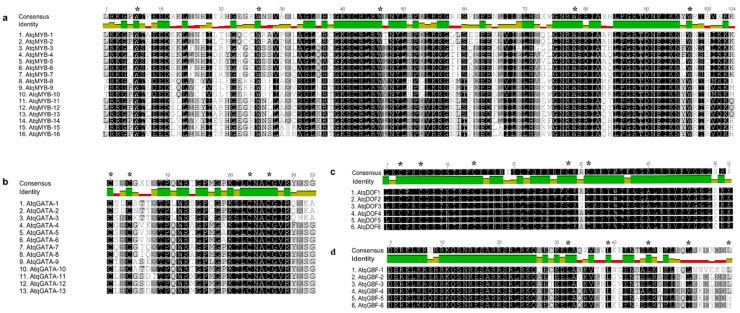
Comparison of amino acid motifs of the MYB, GATA, DOF and GBF transcription factors in *A. tequilana*. (**a**) R2R3 motif of the MYB family, (**b**,**c**) zinc finger motif of the GATA and DOF family and (**d**) the basic leucine zipper (bZIP) of the GBF family. Asterisks (*) indicate tryptophan (W), cysteine (C) and leucine (L) conserved residues that are crucial for the MYB, GATA/DOF and GBF function, respectively. Graphs above alignments indicate highly conserved residues shown as dark green.

**Figure 6:**
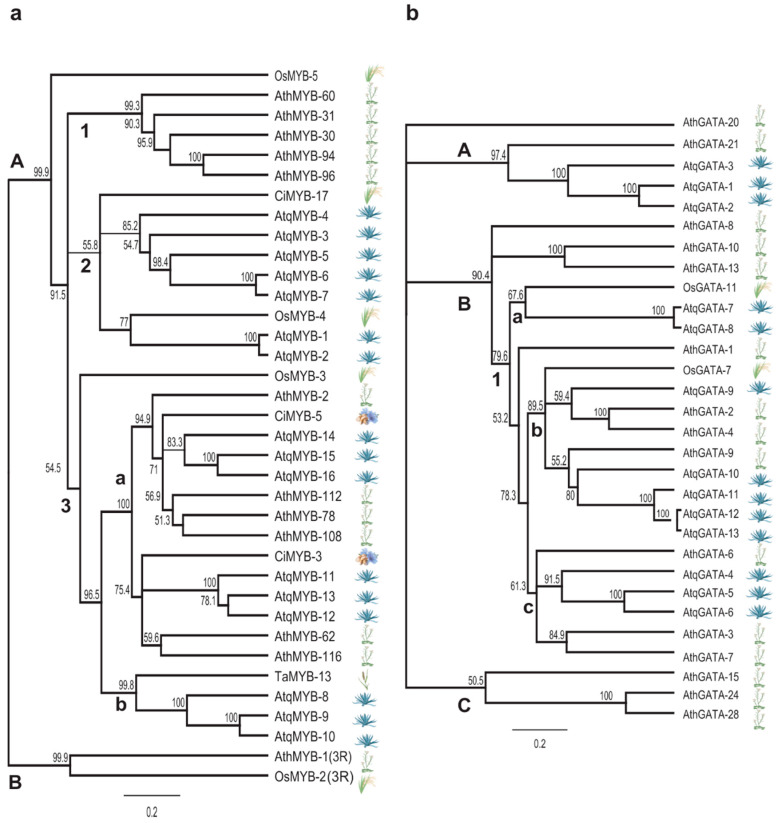

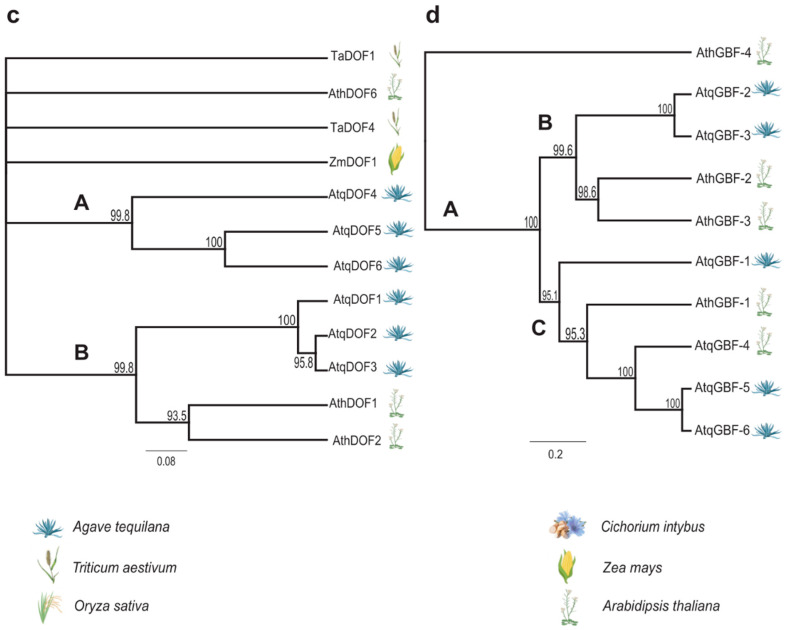
(**a**–**d**) Relationships between amino acid sequences of the (**a**) MYB, (**b**) GATA, (**c**) DOF and (**d**) GBF transcription factors from *A. tequilana* and other plant species. Letters indicate different clades and numbers indicate Bootstrap analysis values. Phylogenetic analyses were carried out using complete amino acid sequences and considering all sites including gaps/missing information based on the UPGMA method and the Jukes-Cantor genetic distance model. Symbology represents the species analyzed.

Based on the motifs identified in the promoter regions of the *A. tequilana* PGHF32 genes and previous reports of transcription factors that regulate PGHF32 in other plant species, a search of the *A. tequilana* transcriptome database was carried out in order to obtain coding sequences for MYB, GATA, DOF and GBF type transcription factors and complete cDNAs could be retrieved for 16 MYB, 13 GATA, 6 DOF and 6 GBF type proteins. Complete cDNA sequences were translated in silico to obtain predicted amino acid sequences for each gene and analyzed to confirm that the conserved domains expected for each type of transcription factor were present (Figure 5). As can be observed, the expected domains were identified for all predicted proteins. To further classify the identified transcription factors within each gene family, dendrograms were produced by aligning the *A. tequilana* transcription factor amino acid sequences with representative members of the same transcription factor gene families from other plant species (Figure 6).

For MYB-type transcription factors, the R1R2R3 type MYB proteins (mainly involved in cell cycle regulation) [41] and the R2R3 type MYB proteins are clearly separated in clades A and B, respectively. Clade A is divided into 3 subclades (A-1, A-2, and A-3) (Figure 6a). Clade A-1 comprises only MYB-type transcription factors from *A. thaliana.* In clade A-2, 7 *A. tequilana* MYB are grouped together with MYB from *C. intybus* and rice. In subclade A-3a, 6 putative MYB proteins from *A. tequilana* were grouped with the MYB from *A. thaliana* and *C. intybus*. In subclade A-3b, 3 MYB proteins from *A. tequilana* are grouped together with TaMYB-13 from *T. aestivum*. OsMYB-3 and 5 did not group within subclade A-1, 2 or 3, although they fall within clade A.

For GATA-like transcription factors, 3 major clades can also be distinguished (Figure 6b). In clade A, AthGATA21 from *A. thaliana* proteins is grouped with three *A. tequilana* GATA proteins (AtqGATA-1, AtqGATA-2 and AtqGATA-3). Clade B contains several subclades, B-1a, B-1b and B-1c. Subgroup B-1 contains AtqGATA-7 and AtqGATA-8 from *A. tequilana* and OsGATA-11 from rice. In subgroup B-1b, 4 *A. tequilana* proteins group most closely with the *A. thaliana* protein AthGATA-9, whereas AtqGATA-9 is grouped most closely with *A. thaliana* proteins AthGATA-2 and 4 shown to respond to abiotic stress and light and is involved in the regulation of the circadian cycle, respectively [42,43]. In subclade B-1c, 3 *A. tequilana* proteins are grouped with proteins from *A. thaliana.* Clade C contains only GATA proteins from *A. thaliana*.

The 6 DOF type proteins (Figure 6c) identified for *A. tequilana* were compared to DOF proteins from wheat, *A. thaliana* and maize and two main clades, A and B, can be distinguished. In clade A, *A. tequilana* DOF proteins form a discrete group. In clade B, *A. tequilana* proteins also form a discrete group but are associated with DOF proteins from *A. thaliana,* which correspond to transcription factors involved in regulation of sucrose metabolism and light and /or show temperature dependent regulation [44,45]. *A. tequilana* proteins did not group with the maize and wheat proteins included in the dendrogram, also known to be involved in regulation of sucrose metabolism and light induction [27,46] or with AthDOF6.

For members of the GBF family, three main clades were suggested (Figure 6d). Clade A contains only AthGBF-4, which differs from the rest of the Arabidopsis GBFs and is unable to form homodimers [47]. In clade B, AthGBF-2 and AthGBF-3 from *A. thaliana* are grouped together with the AtqGBF-2 and AtqGBF-3 sequences from *A. tequilana* and in clade C, AthGBF-1 from *A. thaliana* is grouped together with four proteins from *A. tequilana*.

### 3.4. In Silico Expression Patterns of MYB, GATA GBF and DOF Transcription Factors Identified for A. tequilana

**Figure 7:**
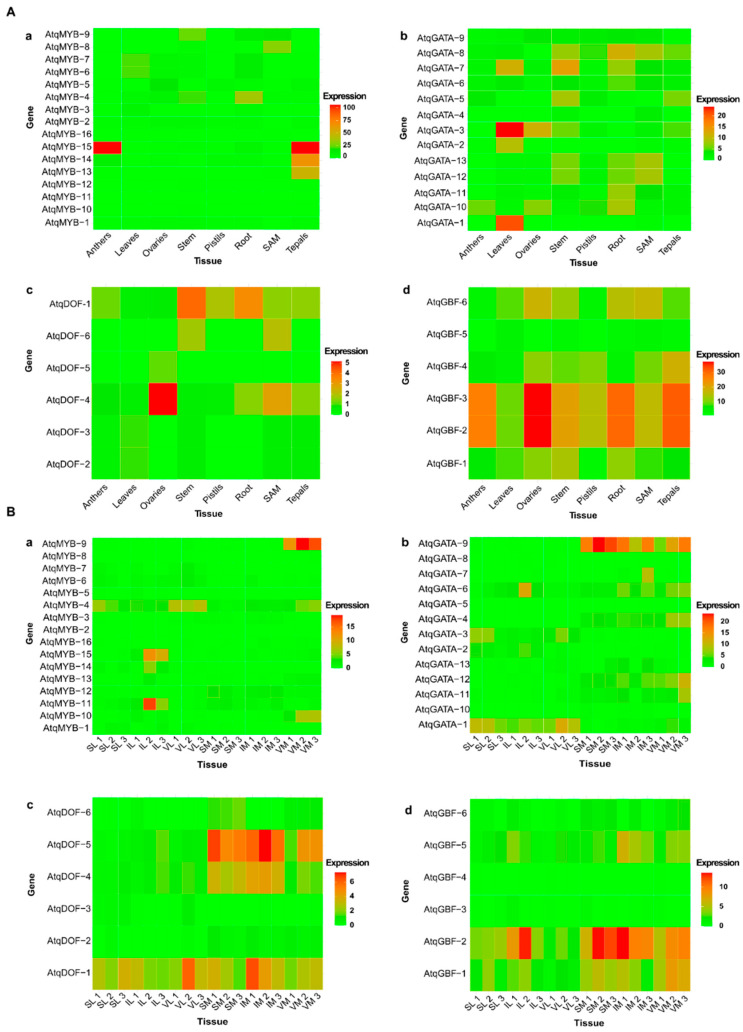
Heat diagrams of in silico gene expression patterns of (**a**) MYB genes, (**b**) GATA genes, (**c**) DOF genes and (**d**) GBF genes. (**A**) vegetative and floral tissues from *A. tequilana* plants as indicated on the x-axis, (2 different plants sampled for each tissue) and (**B**) during the vegetive to reproductive transition as indicated on the x-axis, (3 different plants sampled for each tissue) in *A. tequilana* plants. Red indicates the highest number of transcripts (expression) whereas green indicates the lowest number of transcripts (expression). VL, vegetative leaf, SL, sunken leaf, IL, inflorescence leaf, VM, vegetative shoot apical meristem, SM, sunken shoot apical meristem and IM, inflorescence meristem.

In order to identify candidate transcription factors that could be responsible for the light- or tissue-specific modes of regulation of PGHF32 suggested by the promoter motif analysis and confirmed for leaf tissue by the qRT-PCR analysis, transcriptome databases were analyzed to determine the in silico expression patterns for each of the transcription factors described above (Figure 7). As can be observed, individual transcription factor-encoding genes showed distinct tissue expression patterns (Figure 7A,B). Specific genes for each selected transcription factor showed specific or very strong expression in anthers, leaves or ovaries, as in the case of AtqMYB-15, AtqGATA-1, Atq-GBF-2 and *3* and AtqDOF-4, whereas in contrast other genes showed null or very low expression in all tissues tested, such as AtqMYB-1, 10, 11 and 12, AtqGATA-4 and 9, AtqGBF-5 and AtqDOF-5 (Figure 7A). Figure 7B shows heatmaps of differential expression patterns in leaves and shoot apical meristem (SAM) tissue of the selected transcription factors during the vegetative to reproductive transition in *A. tequilana* and patterns specific to leaf (AtqGATA-1) or SAM (AtqGATA-9) tissue can be observed and for a specific tissue at a specific stage of the transition, for example, leaf tissue in the vegetative and sunken stages (AtqMYB-4) and SAM tissue at the vegetative stage (AtqMYB-9). In the case of the DOF genes, a pattern specific to meristem tissue at all developmental stages during the reproductive transition was observed for AtqDOF-4 and 5, whereas AtqDOF-1 was constitutively expressed at all developmental stages and AtqDOF-2, 3 and 6 showed null or very low levels of expression. AtqGBF-2 and 3 show expression across all tissues tested but higher expression in floral and root tissue and AtqGBF-1, 2 and 5 are expressed at all stages and in both leaf and meristem tissue during the reproductive transition.

## 4. Discussion

Searches of the *A. tequilana* genome database (currently under development at the Advanced Genome Unit at Cinvestav, Mexico) allowed us to identify the genes belonging to PGHF32 in this species. In addition to genes previously identified using RNAseq data [20,21], 3 new genes closely related to genes determined to encode vacuolar invertases were found and tentatively named *AtqVinv-3, 4* and *5,* although confirmation of invertase activity has not yet been carried out. Genome analysis failed to identify candidates for 6-SFT type fructosyltransferase encoding genes, supporting the results from extensive RNAseq analysis in *A. tequilana*, *A. striata*, and *A. victoria-reginae,* where no transcripts encoding putative 6-SFT type enzymes have been found [20]. The complex agavin structures of *A. tequilana* [15] indicate that at least 4 enzyme activities are necessary for their synthesis: 1-SST, 1-FFT, 6G-FFT and 6-SFT. The lack of a separate 6-SFT encoding gene suggests that one of the other enzymes may carry out multiple activities, as has been reported for onion and asparagus, where a bifunctional 6G-FFT/1-FFT enzyme has been described [48,49]. The gene structures for *A. tequilana* PGHF32 are consistent with those described previously by Cortés-Romero et al. 2012 [37] for *Atq1SST-1* and *2, Atq6GFFT-1* and *2, AtqCwinv-1* and *AtqVinv-1*. For the first time, the gene structures for *Atq1SST-3, AtqVinv-2, 3, 4* and *5, Atqinv1* and *2, AtqCwinv-2* and *AtqFEH-1, 2, 3* and *4* are described. A third Cwinv gene (*AtqCwinv-3)* had been identified based on transcriptome data; however, *AtqCwinv-2* and *AtqCwinv-3* mapped to the same genome location, suggesting that the distinct transcripts identified previously are encoded by different alleles at the same locus.

All *A. tequilana* FT and Vinv encoding genes conserve the 8 exon/7 intron structure, whereas the Cwinv encoding genes and *Atqinv1* show a 7 exon/6 intron pattern where exons III and IV are fused. *Atqinv2* has a unique structure with a fusion of exons III and IV and V and VI. *Atqinv1* and *Atqinv2* have not yet been characterized in detail, and although they group with vacuolar invertases from other species (*A. deserti*, rice and maize), their position on the dendrogram and in a previous report indicates that although they are members of PGHF32, their putative function is unclear [20]. They may enter the category of defective invertases that have altered enzyme activities and putative regulatory functions [50]. All the *A. tequilana* FEH encoding genes lack the 9 nucleotide mini-exon characteristic of PGHF32 due to the fusion of exons II and III. In addition, *AtqFEH-3* lacks the mini-exon but also carries an extended first intron and fusion of exons III and IV to produce a 6 exon/5 intron pattern. Introns 2 and 3 are shown to be most variable in size and the relocation of the mini-exon closer to exon III in structures b′ and c may suggest a possible inversion of the region covering introns 1 and 2.

Comparison of PGHF32 gene structures from a selection of different plant species uncovered 7 different exon/intron patterns, which, with the exception of the 9/8 pattern, were shared across the taxonomic groups. However, superimposing the exon/intron patterns on a dendrogram showing the relationships between gene types revealed a very good correlation. The exon/intron structures observed for fructan-producing species also support the hypothesis that fructosyltransferases evolved from vacuolar invertases and that fructan exohydrolases evolved from cell wall invertases. For example, fructosyltransferases and vacuolar invertases from *A. tauschii*, wheat and barley all share either 4/3 or 3/2 exon/intron structures. In comparison, all cell wall invertases from these species have a 7/6 pattern and fructan exohydrolases have a mixture of 9/8 and 7/6 patterns. Similarly for *A. tequilana* and *A. officinalis*, all fructosyltransferase and vacuolar invertase genes have and 8/7 structures whereas cell wall invertase genes in common with *A. tauschii*, wheat and barley have the 7/6 structure and FEH genes have mainly a 6/5 structure.

**Figure 8:**
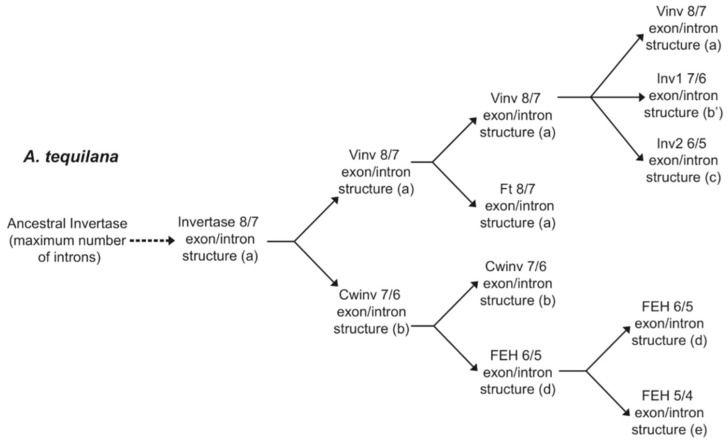
Diagram showing a possible scenario to describe how gene duplication and intron loss have produced the exon/intron patterns observed for the *A. tequilana* PGHF32 genes. Exon/intron patterns and structures a, b, b′, c, d and e are those described in Figure 1.

Gene function, however, is not directly related to the evolution of the exon/intron patterns since, for example, all *A. tequilana* FT and vacuolar invertases share the same pattern but have clearly different enzyme activities. The only case where a good correlation between function and structure was observed in the case of the *A. tequilana* FEH genes where all have lost the second intron and the mini-exon is fused to exon III, whereas the Cwinv genes maintain intron 2 and the mini-exon. The presence or absence of the mini-exon is also relevant for the regulation of PGHF32 genes since it has been shown that this 9 nucleotide exon, which encodes a highly conserved amino acid motif, can undergo differential splicing under specific conditions leading to regulation at the post transcriptional level [51]. In general, the modifications in exon/intron patterns can be explained by progressive loss of introns and in some cases (shown in red in Figure 2) can be directly attributed to loss of intron 2 and mini-exon fusion. A possible scenario for the progression of intron loss for *A. tequilana* PGHF32 is shown in Figure 8. As genome data become available, it will be interesting to extend comparisons of PGHF32 gene structure in both fructan synthesizing and non-synthesizing species and determine whether structural changes are related to gene function and regulation.

RNAseq data has shown that PGHF32 genes are differentially regulated in a tissue-specific manner and during different stages of development [20,21]. Individual genes are also regulated by stress and hormone treatments [23,24]. These results are consistent with reports for PGHF32 members in many other species [26,28,31]. The availability of genome data allowed investigation for the first time of the promoter regions of the *A. tequilana* PGHF32 genes in order to identify putative regulatory motifs responsible for their transcriptional regulation under distinct conditions and tissue types. Perhaps surprisingly, the most abundant motifs were found to be associated with light regulation; however, this may reflect the importance of both fructan and Crassulacean Acid Metabolism (CAM) for *Agave* species. In *Agaves,* only very low levels of starch, located mainly in the guard cells, are found in photosynthetically active tissues, whereas fructooligosaccharides are abundant [22,52] and may be transported to sink tissues [5]. This implies a need for close coordination between CAM photosynthesis cycles and fructan metabolism. Since few plant species combine both fructan and CAM metabolism [14,32], *Agaves* represent an important model for determining the coordinated regulation of these processes that also have significance for the capacity of *Agave* species to tolerate arid environments. One surprising aspect of the light regulatory motifs identified was the low number or absence of GBF motifs in the *A. tequilana* PGHF32 promoters since GBF has been shown in other species to be essential components of light regulation in association with the GATA motif [53]. It will therefore be of interest to explore in detail the light regulatory mechanisms in *A. tequilana*.

Other abundant motifs were consistent with previous reports of factors affecting the expression of genes encoding enzymes involved in fructan metabolism in both *Agaves* and other plant species [25,26,27,28,29,30]. The DOF and YACT motifs associated with carbohydrate metabolism and photosynthetically active tissues, respectively, also support a strong coordination between fructan and CAM metabolism [54,55,56]. Regulation by phytohormones, such as cytokinins and especially gibberellins, is also consistent with previous reports on differential gene expression profiles during the vegetative to reproductive transition [29], and significant numbers of motifs associated with floral tissue (pollen and embryogenesis) and root tissue all confirm previous reports for PGHF32 genes in *A. tequilana* [28]. MYB transcription factors have been shown to play essential roles in the regulation of fructan metabolism genes in *C. intybus* and in response to light, biotic stress and sucrose or phytohormone signaling in *A. thaliana* [26,31,57]. However, although MYB motifs associated with dehydration were identified in all *A. tequilana* PGHF32 genes, they were less abundant than those associated with the other transcription factors described above. The dendrograms comparing *A. tequilana* transcription factor proteins with those of other species in some cases showed very low bootstrap values, indicating that for these clades or branches the topology is less robust. This may reflect the observation that although the characteristic domains for each transcription factor are highly conserved, as shown in Figure 5, these proteins are quite heterogeneous in the surrounding sequences. The inclusion of greater numbers of proteins from a wider variety of species in the comparisons should lead to dendrograms with more robust topologies.

The qRT-PCR analyses experimentally confirm that the selected group of PGHF32 genes are regulated by light and show tissue-specific patterns of regulation in leaves. Although all the PGHF32 genes shared elements for light- and tissue-specific regulation, they did not all show the same regulatory responses. Whereas the FT encoding genes (*Atq1SST-1, Atq6GFFT1* and *2*) and the vacuolar invertase gene *AtqVinv-1* all showed clear effects of light regulation, the exohydrolase gene *AtqFEH-4* was expressed at a low level and showed a slight induction under darkness. When green and white leaf tissues were compared, the 3 FT encoding genes shared the same pattern of high expression in white leaf tissue in comparison to green leaf tissue, whereas *AtqVinv-1* was repressed in white tissue in comparison to green tissue. On the other hand, *AtqFEH-4* was slightly induced in white tissue compared to green tissue. These results reflect the complexity and precision of transcriptional gene regulation accomplished by the interactions of a battery of transcription factors that respond to localized cellular conditions or metabolites, as well as environmental factors, such as light or water stress.

**Figure 9:**
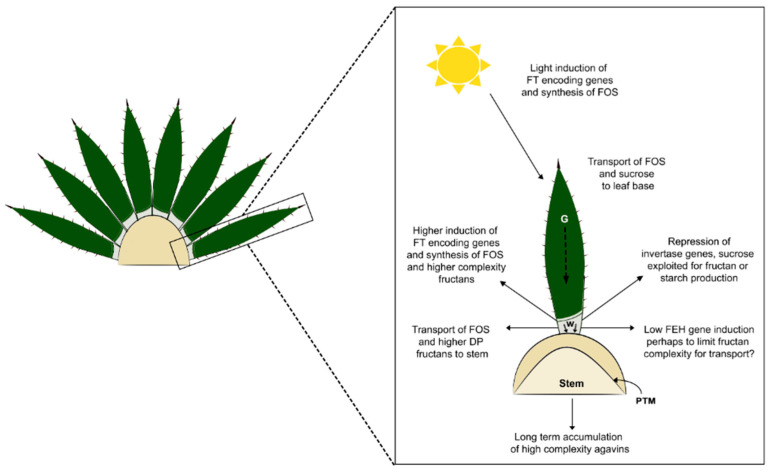
Model for the integration of light and leaf specific regulation of PGHF32 members into overall carbohydrate metabolism in *A. tequilana*. FT, fructosyltransferases, FOS, fructooligosaccharide, DP, degree of polymerization, G, green leaf tissue, W, white leaf tissue, PTM, primary thickening meristem.

Although the qRT-PCR results do not include the whole set of PGHF32 genes, the expression patterns observed are consistent with previous reports on fructan metabolism in *Agave* species, as summarized in Figure 9. Sucrose is synthesized as the product of photosynthesis and can be exploited for the synthesis of fructans in green leaf tissue. Both sucrose and fructans can be mobilized to sink tissues, such as the stem. Sucrose can be used to synthesize more complex fructans in the leaf base and stem tissue or converted to starch in the peripheral growth meristem (to be utilized for growth and expansion of the stem). It is consistent that invertase activity is lower in white tissue so that sucrose can be used by fructosyltransferases and starch synthesizing enzymes. It is possible that a low level of fructo exohydrolase activity is needed to ensure that mainly fructooligosaccharides are present in leaf base tissue and can be transported to the stem where they are then converted into more complex fructan polymers for long-term storage.

The in silico expression patterns determined for the transcription factor genes are also consistent with putative roles in the regulation of PGHF32 genes in *A. tequilana.* Specific GATA encoding genes are highly expressed in leaf tissue in general and in SAM tissue at different stages of the vegetative to reproductive transition. Specific DOF genes are strongly expressed in floral and stem tissue and in SAM tissue throughout the vegetative to reproductive transition, perhaps reflecting changes in carbohydrate metabolism in these actively growing tissues. Specific MYB genes are also strongly expressed in floral tissues, leaves and in vegetative SAM tissue. GBF transcription factors showed strong expression in floral and root tissues, consistent with patterns of PGHF32 expression.

In order to isolate and characterize in detail the FT encoding genes involved in the regulation of *A. tequilana* PGHF32, it is essential to identify the best candidates of these multigene families based on their phylogenetic relations with previously characterized genes in other plant species and on their expression patterns in *A. tequilana*. For example, AtqGATA-1 and 3 are strongly expressed in leaf tissue and are found in a clade containing genes from *A. thaliana* known to be involved in light and circadian regulation. AtqGATA-1 and 3 are therefore strong candidates for participating in the light regulation of the PGHF32 genes. Knowledge of in silico transcription levels in combination with the full-length cDNAs used to produce the dendrograms are useful tools for the cloning of the candidate TF genes for further analysis.

## 5. Conclusions

Based on genome analysis, members of *A. tequilana* PGHF32 previously identified by RNAseq were confirmed with 3 newly identified Vinv genes completing the family. The lack of a separate 6-SFT encoding gene was also confirmed. Analysis of gene structures for all *A. tequilana* PGHF32 genes revealed exon/intron patterns consistent with other plant species, although specific for *Agaves* and supports the hypothesis of evolution of FT from vacuolar invertases and FEH from cell wall invertases. In addition, the presence of distinct exon/intron patterns can be attributed to the loss of specific introns and fusion of surrounding exons. Analysis of promoter motifs suggests that PGHF32 genes in *A. tequilana* are strongly regulated by light and are tissue specific. In silico expression analysis, in combination with phylogenic analysis comparing selected *A. tequilana* transcription factor genes with those of other plant species, identified candidate genes for future detailed characterization of the regulatory mechanisms associated with PGHF32.

## Data Availability

*A. tequilana* draft genome (Herrera-Estrella et al. unpublished), permission to access the data prior to publication can be arranged by directly contacting Dr. Alfredo Herrera-Estrella: (alfredo.herrera@cinvestav.mx) or Dr. Selene Fernández Valverde: (selene.fernandez@cinvestav.mx).

## Supplementary Materials

The following supporting information can be downloaded at: https://www.mdpi.com/article/10.3390/plants11162153/s1, Figure S1: *Agave tequilana* leaf showing the photosynthetically active aerial green tissue and the non-photosynthetically active basal white tissue; Figure S2: Example of the pattern of abundance of regulatory motifs found in *Agave tequilana* PGHF32 promoter regions based on *A. tequilana* FEH genes; Figure S3: qRT-PCR analysis of PGHF32 genes in green (G) and white (W) leaf regions; Table S1: List of species and accession numbers for PGHF32 sequences used in alignments and phylogenetic analyses; Table S2: List of species and accession numbers for all sequences of transcription factors MYB, GATA, DOF and GBF used in alignments and phylogenetic analyses; Table S3: Gene structure conformation of PGHF32 genes in monocotyledonous and dicotyledonous species; Table S4: Nucleotide conformation in exons and introns of genes encoding the PGHF32 enzymes in *Agave tequilana*.
