# Supplementary material for: Evidence for Light and Tissue Specific Regulation of Genes Involved in Fructan Metabolism in Agave tequilana
Source: Plants (Basel). 2022 Aug 19;11(16):2153. doi: 10.3390/plants11162153 (PMC9412663; doi:10.3390/plants11162153)
Supplement: Supplementary file 1 [file plants-11-02153-s001.zip › Supplementary figure captions corrected.pdf]

Supplementary Figure S1. *Agave tequilana* leaf showing the photosynthetically active aerial green tissue and the non-photosynthetically active basal white tissue.

Supplementary Figure S2. Example of the pattern of abundance of regulatory motifs found in *Agave tequilana* PGHF32 promoter regions based on *A. tequilana* FEH genes.

Supplementary Figure S3. qRT-PCR analysis of PGHF32 genes in green (G) and white (W) leaf regions. Error bars indicate the standard deviation. The genes analyzed are indicated above each graph.
