# Supplementary material for: Evidence for Light and Tissue Specific Regulation of Genes Involved in Fructan Metabolism in Agave tequilana
Source: Plants (Basel). 2022 Aug 19;11(16):2153. doi: 10.3390/plants11162153 (PMC9412663; doi:10.3390/plants11162153)
Supplement: Supplementary file 1 [file plants-11-02153-s001.zip › Supplementary Table S1 .pdf]

Supplementary Table S 1. List of species, *Agave tequilana*, asparagus (*Asparagus officinalis)*, goatgrass *(Aegilops tauschii*)*,* wheat (*Triticum aestivum)*, barley (*Hordeum vulgare)*, *Arabidopsis thaliana*, carrot (*Daucus carota*), beetroot (*Beta vulgaris*), maize (*Zea mays*) and rice (*Oryza sativa*), and accession numbers for PGHF32 sequences used in alignments and phylogenetic analyses.

**Class**	**Species**	**Accession**	**Code**
**Monocotyledon**	*A. tauschii*	XM_020304935.1	Ata1SST-like
**Monocotyledon**	*A. tauschii*	XM_020334947.1	Ata1FFT-like
**Monocotyledon**	*A. tauschii*	XM_020301133.1	Ata6SFT-like
**Monocotyledon**	*A. tauschii*	XM_020321719.1	AtaInvlike1-like
**Monocotyledon**	*A. tauschii*	XM_020307765.1	Ata1FEH-like
**Monocotyledon**	*A. tauschii*	XM_020326258.1	AtaVinv1-like
**Monocotyledon**	*A. tauschii*	XM_020327311.1	AtaCwinv1-like
**Monocotyledon**	*H. vulgare*	AK354338	Hv1FFT
**Monocotyledon**	*H. vulgare*	AK356300	Hv6FEH
**Monocotyledon**	*H. vulgare*	JQ411255	HvInv1
**Monocotyledon**	*H. vulgare*	AK357105.1	HvCwinv1-like
**Monocotyledon**	*H. vulgare*	AJ605333	Hv1FEH
**Monocotyledon**	*H. vulgare*	JQ411256	HvInv2
**Monocotyledon**	*H. vulgare*	JQ411252.1	Hv1SST
**Monocotyledon**	*H. vulgare*	X83233	Hv6SFT
**Monocotyledon**	*O. sativa*	XM_015781448.2	OsInv2
**Monocotyledon**	*O. sativa*	XM_015770302.2	OsInv3

**Monocotyledon**	*O. sativa*	XM_015766627.2	OsInv4
**Monocotyledon**	*O. sativa*	XM_015778048.2	OsCwinv2
**Monocotyledon**	*O. sativa*	XM_015766627.1	OsCwinv4
**Monocotyledon**	*O. sativa*	XM_015778898.2	OsCwinv6
**Monocotyledon**	*O. sativa*	XM_015769787.2	OsCwinv1
**Monocotyledon**	*O. sativa*	XM_015757090.2	OsInv1
**Monocotyledon**	*Z. mays*	NM_001111899.2	ZmCwinv1
**Monocotyledon**	*Z. mays*	NM_001112126.2	ZmCwinv2
**Monocotyledon**	*Z. mays*	NM_001371613.1	ZmCwinv3
**Monocotyledon**	*Z. mays*	XM_020547998.1	ZmCwinv4
**Monocotyledon**	*Z. mays*	XM_008670214.3	ZmInv1
**Monocotyledon**	*Z. mays*	XM_020545958.1	ZmInv2
**Monocotyledon**	*Z. mays*	NM_001372181.1	Zm6,1FEH
**Monocotyledon**	*T. aestivum*	AJ508387	Ta1FEHw2
**Monocotyledon**	*T. aestivum*	AB088409	Ta1FFTA
**Monocotyledon**	*T. aestivum*	AB088410	Ta1FFTB
**Monocotyledon**	*T. aestivum*	AB029888	Ta1SST
**Monocotyledon**	*T. aestivum*	AM075205	Ta6FEH
**Monocotyledon**	*T. aestivum*	AB029887	Ta6SFT
**Monocotyledon**	*T. aestivum*	AB089269	Ta6,1FEH
**Monocotyledon**	*T. aestivum*	AJ516025	Ta1FEHw1

**Monocotyledon**	*T. aestivum*	AB089271	Ta6KEHw1
**Monocotyledon**	*T. aestivum*	AB089270	Ta6KEHw2
**Monocotyledon**	*T. aestivum*	AJ564996	Ta1FEHw3
**Monocotyledon**	*T. aestivum*	AJ635225	TaInv1-like
**Monocotyledon**	*T. aestivum*	AB196522	TaCwinv1-like
**Monocotyledon**	*T. aestivum*	TraesCS2A02G295400.1 (Ensembl plants)	TaCwinv2-like
**Monocotyledon**	*T. aestivum*	TraesCS2B02G311900.1 (Ensembl plants)	TaCwinv3-like
**Monocotyledon**	*T. aestivum*	TraesCS7D02G010000.2 (Ensembl plants)	TaVinv2-like
**Monocotyledon**	*T. aestivum*	TraesCS4A02G484800.1 (Ensembl plants)	TaVinv3-like
**Monocotyledon**	*A. officinalis*	AB115555<br>XM_020385299.1	Ao1SST
**Monocotyledon**	*A. officinalis*	AB115554.1<br>XM_020421252.1	Ao1FFT1
**Monocotyledon**	*A. officinalis*	AB084283<br>XM_020390805.1	Ao6GFFT
**Monocotyledon**	*A. officinalis*	AB195641<br>XM_020397679.1	Ao6FEH
**Monocotyledon**	*A. officinalis*	AB244731<br>XM_020397667.1	AoCwinv1-like
**Monocotyledon**	*A. officinalis*	XM_020390005.1	AoCwinv2-like
**Monocotyledon**	*A. tequilana*	EU026119<br>ON553358	Atq1FFT<br>Gene Atq1FFT
**Monocotyledon**	*A. tequilana*	JN790053<br>ON553353	Atq1SST-1<br>Promoter Atq1SST-1
**Monocotyledon**	*A. tequilana*	JN790054<br>ON553354	Atq1SST-2<br>Promoter Atq1SST-2
**Monocotyledon**	*A. tequilana*	MK251792<br>ON553355	Atq1SST-3<br>Gene Atq1SST-3
**Monocotyledon**	*A. tequilana*	JN790055<br>ON553356	Atq6GFFT-1<br>Promoter Atq6GFFT-1
**Monocotyledon**	*A. tequilana*	JN790056<br>ON553357	Atq6GFFT-2<br>Promoter Atq6GFFT-2

**Monocotyledon**	*A. tequilana*	JN790058<br>ON553359	AtqVinv1<br>Promoter AtqVinv1
**Monocotyledon**	*A. tequilana*	GAHU01007017<br>ON553360	AtqVinv2<br>Gene AtqVinv2
**Monocotyledon**	*A. tequilana*	ON553350<br>ON553369	AtqVinv3-like<br>Gene AtqVinv3-like
**Monocotyledon**	*A. tequilana*	ON553351<br>ON553370	AtqVinv4-like<br>Gene AtqVinv4-like
**Monocotyledon**	*A. tequilana*	ON553352<br>ON553371	AtqVinv5-like<br>Gene AtqVinv5-like
**Monocotyledon**	*A. tequilana*	GAHU01060462<br>ON553361	AtqInv1<br>Gene AtqInv1
**Monocotyledon**	*A. tequilana*	KR138450<br>ON553362	AtqInv2<br>Gene AtqInv2
**Monocotyledon**	*A. tequilana*	JN790057<br>ON553363	AtqCwinv-1<br>Gene AtqCwnv-1
**Monocotyledon**	*A. tequilana*	GAHU01053508<br>ON553364	AtqCwinv-2<br>Gene AtqCwinv-2
**Monocotyledon**	*A. tequilana*	KR138454<br>ON553365	AtqFEH-1<br>Gene AtqFEH-1
**Monocotyledon**	*A. tequilana*	GAHU01085730<br>ON553366	AtqFEH-2<br>Gene AtqFEH-2
**Monocotyledon**	*A. tequilana*	KR138455<br>ON553367	AtqFEH-3<br>Gene AtqFEH-3
**Monocotyledon**	*A. tequilana*	GAHU01012712<br>ON553368	AtqFEH-4<br>Gene AtqFEH-4
**Dicotyledon**	*A. thaliana*	(TAIR: AT1G62660)<br>NM_104943	AthVinv1
**Dicotyledon**	*A. thaliana*	(TAIR: AT1G12240)<br>NM_101096	AthVinv2
**Dicotyledon**	*A. thaliana*	(TAIR:AT3G13790)<br>NM_112232	AthCwinv1
**Dicotyledon**	*A. thaliana*	(TAIR:AT3G52600)<br>NM_115120	AthCwinv2
**Dicotyledon**	*A. thaliana*	(TAIR:AT2G36190)<br>NM_129177	AthCwinv4
**Dicotyledon**	*A. thaliana*	(TAIR:AT3G13784)<br>NM_112231	AthCwinv5
**Dicotyledon**	*A. thaliana*	(TAIR:AT1G55120)<br>NM_104385	AthCwinv3 (6-FEH)
**Dicotyledon**	*A. thaliana*	(TAIR:AT5G11920)<br>NM_121230.3	AthCwinv6 (6,1-FEH)

**Dicotyledon**	*B. vulgaris*	XM_010687077.2	Bv6FEH
**Dicotyledon**	*B. vulgaris*	XM_010677873.2	BvVinv
**Dicotyledon**	*B. vulgaris*	XM_010671083.2	BvCwinv
**Dicotyledon**	*D. carota*	XM_017396738.1	DcVinv1-like
**Dicotyledon**	*D. carota*	XM_017391458.1	DcVinv2-like
**Dicotyledon**	*D. carota*	XM_017389711.1	DcVinv3-like
**Dicotyledon**	*D. carota*	XM_017401429.1	DcCwinv2-like
**Dicotyledon**	*D. carota*	XM_017373748.1	DcCwinv1-like
**Dicotyledon**	*D. carota*	XM_017362189.1	DcCwinv3-like
