# Supplementary material for: Evidence for Light and Tissue Specific Regulation of Genes Involved in Fructan Metabolism in Agave tequilana
Source: Plants (Basel). 2022 Aug 19;11(16):2153. doi: 10.3390/plants11162153 (PMC9412663; doi:10.3390/plants11162153)
Supplement: Supplementary file 1 [file plants-11-02153-s001.zip › Supplementary Table S2 .pdf]

Supplementary Table S 2. List of species, *Agave tequilana*, asparagus (*Asparagus officinalis)*, goatgrass *(Aegilops tauschii*), wheat (*Triticum aestivum)*, barley (*Hordeum vulgare)*, *Arabidopsis thaliana*, carrot (*Daucus carota*), beetroot (*Beta vulgaris*), maize (*Zea mays*) and rice (*Oryza sativa*), and accession numbers for all sequences of transcription factors MYB, GATA, DOF and GBF used in alignments and phylogenetic analyses.

Class	Species	Accession	Transcription factor	Code
**Monocotyledon**	*T. aestivum*	JF288934	MYB-13	TaMYB-13
**Monocotyledon**	*O. sativa*	BAA23339.1	MYB-3	OsMYB-3
**Monocotyledon**	*O. sativa*	BAA23340.1	MYB-4	OsMYB-4
**Monocotyledon**	*O. sativa*	BAA23341.1	MYB-5	OsMYB-5
**Dicotyledon**	*C. intybus*	KY354366.1	MYB-3	CiMYB-3
**Dicotyledon**	*C. intybus*	KY354368.1	MYB-5	CiMYB-5
**Dicotyledon**	*C. intybus*	KY354369.1	MYB-17	CiMYB-17
**Dicotyledon**	*A. thaliana*	AT2G47190.1	MYB-2	AtMYB-2
**Dicotyledon**	*A. thaliana*	AT3G28910.1	MYB-30	AtMYB-30
**Dicotyledon**	*A. thaliana*	AT1G74650.1	MYB-31	AtMYB-31
**Dicotyledon**	*A. thaliana*	AT1G08810.1	MYB-60	AtMYB-60
**Dicotyledon**	*A. thaliana*	AT1G68320.1	MYB-62	AtMYB-62
**Dicotyledon**	*A. thaliana*	AT3G47600.1	MYB-94	AtMYB-94
**Dicotyledon**	*A. thaliana*	AT5G62470.1	MYB-96	AtMYB-96
**Dicotyledon**	*A. thaliana*	AT5G49620.1	MYB-78	AtMYB-78
**Dicotyledon**	*A. thaliana*	AT3G06490.1	MYB-108	AtMYB-108
**Dicotyledon**	*A. thaliana*	AT1G48000.1	MYB-112	AtMYB-112
**Dicotyledon**	*A. thaliana*	AT1G25340.1	MYB-116	AtMYB-116

**Dicotyledon**	*A. thaliana*	AT4G32730.1	MYB-1(3R)	AtMYB-1(3R)
**Monocotyledon**	*O. sativa*	XM_015761007	MYB-2(3R)	OsMYB-2(3R)
**Monocotyledon**	*A.tequilana*	ON553309	MYB-1	AtqMYB-1
**Monocotyledon**	*A.tequilana*	ON553310	MYB-2	AtqMYB-2
**Monocotyledon**	*A.tequilana*	ON553311	MYB-3	AtqMYB-3
**Monocotyledon**	*A.tequilana*	ON553312	MYB-4	AtqMYB-4
**Monocotyledon**	*A.tequilana*	ON553313	MYB-5	AtqMYB-5
**Monocotyledon**	*A.tequilana*	ON553314	MYB-6	AtqMYB-6
**Monocotyledon**	*A.tequilana*	ON553315	MYB-7	AtqMYB-7
**Monocotyledon**	*A.tequilana*	ON553316	MYB-8	AtqMYB-8
**Monocotyledon**	*A.tequilana*	ON553317	MYB-9	AtqMYB-9
**Monocotyledon**	*A.tequilana*	ON553318	MYB-10	AtqMYB-10
**Monocotyledon**	*A.tequilana*	ON553319	MYB-11	AtqMYB-11
**Monocotyledon**	*A.tequilana*	ON553320	MYB-12	AtqMYB-12
**Monocotyledon**	*A.tequilana*	ON553321	MYB-13	AtqMYB-13
**Monocotyledon**	*A.tequilana*	ON553322	MYB-14	AtqMYB-14
**Monocotyledon**	*A.tequilana*	ON553323	MYB-15	AtqMYB-15
**Monocotyledon**	*A.tequilana*	ON553324	MYB-16	AtqMYB-16
**Monocotyledon**	*T. aestivum*	AY955493	DOF-1	TaDOF-1
**Monocotyledon**	*T. aestivum*	FJ687390	DOF-4	TaDOF-4
**Monocotyledon**	*Z. mays*	CAA46875	DOF-1	ZmDOF-1
**Dicotyledon**	*A. thaliana*	AT1G51700.1	DOF-1	AthDOF-1
**Dicotyledon**	*A. thaliana*	AT3G21270.1	DOF-2	AthDOF-2

**Dicotyledon**	*A. thaliana*	AT3G45610.1	DOF-6	AthDOF-6
**Monocotyledon**	*A.tequilana*	ON553325	DOF-1	Atq DOF-1
**Monocotyledon**	*A.tequilana*	ON553326	DOF-2	Atq DOF-2
**Monocotyledon**	*A.tequilana*	ON553327	DOF-3	Atq DOF-3
**Monocotyledon**	*A.tequilana*	ON553328	DOF-4	Atq DOF-4
**Monocotyledon**	*A.tequilana*	ON553329	DOF-5	Atq DOF-5
**Monocotyledon**	*A.tequilana*	ON553330	DOF-6	Atq DOF-6
**Dicotyledon**	*A. thaliana*	AT4G36730.1	GBF-1	AthGBF-1
**Dicotyledon**	*A. thaliana*	AT4G01120.1	GBF-2	AthGBF-2
**Dicotyledon**	*A. thaliana*	AT2G46270.1	GBF-3	AthGBF-3
**Dicotyledon**	*A. thaliana*	AT1G03970.1	GBF-4	AthGBF-4
**Monocotyledon**	*A.tequilana*	ON553344	GBF-1	AtqGBF-1
**Monocotyledon**	*A.tequilana*	ON553345	GBF-2	AtqGBF-2
**Monocotyledon**	*A.tequilana*	ON553346	GBF-3	AtqGBF-3
**Monocotyledon**	*A.tequilana*	ON553347	GBF-4	AtqGBF-4
**Monocotyledon**	*A.tequilana*	ON553348	GBF-5	AtqGBF-5
**Monocotyledon**	*A.tequilana*	ON553349	GBF-6	AtqGBF-6
**Monocotyledon**	*O. sativa*	AAP54978	GATA-7	OsGATA-7
**Monocotyledon**	*O. sativa*	XP_015626107	GATA-11	OsGATA-11
**Dicotyledon**	*A. thaliana*	AT3G24050.1	GATA-1	AthGATA-1
**Dicotyledon**	*A. thaliana*	AT2G45050.1	GATA-2	AthGATA-2
**Dicotyledon**	*A. thaliana*	AT4G34680.1	GATA-3	AthGATA-3
**Dicotyledon**	*A. thaliana*	AT3G60530.1	GATA-4	AthGATA-4

**Dicotyledon**	*A. thaliana*	AT3G51080.1	GATA-6	AthGATA-6
**Dicotyledon**	*A. thaliana*	AT4G36240.1	GATA-7	AthGATA-7
**Dicotyledon**	*A. thaliana*	AT3G54810.1	GATA-8	AthGATA-8
**Dicotyledon**	*A. thaliana*	AT4G32890.1	GATA-9	AthGATA-9
**Dicotyledon**	*A. thaliana*	AT1G08000.1	GATA-10	AthGATA-10
**Dicotyledon**	*A. thaliana*	AT2G28340.1	GATA-13	AthGATA-13
**Dicotyledon**	*A. thaliana*	AT3G06740.1	GATA-15	AthGATA-15
**Dicotyledon**	*A. thaliana*	AT2G18380.1	GATA-20	AthGATA-20
**Dicotyledon**	*A. thaliana*	AT5G56860.1	GATA-21	AthGATA-21
**Dicotyledon**	*A. thaliana*	AT3G21175.1	GATA-24	AthGATA-24
**Dicotyledon**	*A. thaliana*	AT4G17570.1	GATA-26	AthGATA-26
**Dicotyledon**	*A. thaliana*	AT1G51600.1	GATA-28	AthGATA-28
**Monocotyledon**	*A.tequilana*	ON553331	GATA-1	AtqGATA-1
**Monocotyledon**	*A.tequilana*	ON553332	GATA-2	AtqGATA-2
**Monocotyledon**	*A.tequilana*	ON553333	GATA-3	AtqGATA-3
**Monocotyledon**	*A.tequilana*	ON553334	GATA-4	AtqGATA-4
**Monocotyledon**	*A.tequilana*	ON553335	GATA-5	AtqGATA-5
**Monocotyledon**	*A.tequilana*	ON553336	GATA-6	AtqGATA-6
**Monocotyledon**	*A.tequilana*	ON553337	GATA-7	AtqGATA-7
**Monocotyledon**	*A.tequilana*	ON553338	GATA-8	AtqGATA-8
**Monocotyledon**	*A.tequilana*	ON553339	GATA-9	AtqGATA-9
**Monocotyledon**	*A.tequilana*	ON553340	GATA-10	AtqGATA-10
**Monocotyledon**	*A.tequilana*	ON553341	GATA-11	AtqGATA-11

**Monocotyledon**	*A.tequilana*	ON553342	GATA-12	AtqGATA-12
**Monocotyledon**	*A.tequilana*	ON553343	GATA-13	AtqGATA-13
