# Supplementary material for: Evidence for Light and Tissue Specific Regulation of Genes Involved in Fructan Metabolism in Agave tequilana
Source: Plants (Basel). 2022 Aug 19;11(16):2153. doi: 10.3390/plants11162153 (PMC9412663; doi:10.3390/plants11162153)
Supplement: Supplementary file 1 [file plants-11-02153-s001.zip › Supplementary table S3.pdf]

Supplementary Table S3. Nucleotide conformation in exons and introns of genes encoding the PGHF32 enzymes in *Agave tequilana*.

Gene	Exon 1	Intron 1	Exon 2	Intron 2	Exon 3	Intron 3	Exon 4	Intron 4	Exon 5	Intron 5	Exon 6	Intron 6	Exon 7	Intron 7	Exon 8
AtqSST-1	279	149	9	1554	382	75	500	84	163	106	236	91	90	130	207
AtqSST-2	279	134	9	1095	382	99	499	67	164	132	236	89	90	184	207
AtqSST-3	281	170	9	1444	382	92	500	81	162	73	237	106	90	93	207
AtqFFT-1	312	161	9	3263	385	705	501	183	162	381	236	91	89	739	214
Atq6GFFT-1	255	160	9	1491	391	75	500	125	163	107	233	539	89	356	244
Atq6GFFT-2	276	161	9	3336	382	95	500	118	162	90	236	91	89	86	215
AtqVinv-1	375	166	9	1821	379	311	494	107	163	124	236	95	89	97	196
AtqVinv-2	369	194	9	2084	383	146	490	93	163	100	236	92	89	93	217
AtqInv1	336	162	9	361	855	2015	154	165	235	91	92	233	213		
AtqInv2	357	443	8	47	833	363	397	95	90	140	213				
AtqCwinv-1	183	1491	9	103	857	101	158	121	242	83	93	106	188		
AtqCwinv-2	189	940	9	113	856	95	158	152	242	88	93	108	187		
AtqFEH-1	201	673	828	228	160	91	248	120	95	109	181				
AtqFEH-2	198	1064	831	256	161	87	243	273	94	119	182				
AtqFEH-3	221	2641	847	78	403	86	95	112	181						
AtqFEH-4	210	3165	846	94	158	112	244	108	95	91	181				
