# Supplementary material for: Evidence for Light and Tissue Specific Regulation of Genes Involved in Fructan Metabolism in Agave tequilana
Source: Plants (Basel). 2022 Aug 19;11(16):2153. doi: 10.3390/plants11162153 (PMC9412663; doi:10.3390/plants11162153)
Supplement: Supplementary file 1 [file plants-11-02153-s001.zip › Supplementary Table S4.pdf]

Supplementary Table S4. Gene structure conformation of PGHF32 genes in monocotyledonous and dicotyledonous species, *Agave tequilana*, asparagus (*Asparagus officinalis)*, goatgrass *(Aegilops tauschii*), wheat (*Triticum aestivum)*, barley (*Hordeum vulgare)*, *Arabidopsis thaliana*, carrot (*Daucus carota*), beetroot (*Beta vulgaris*), maize (*Zea mays*) and rice (*Oryza sativa*). The mini-exon is located in Exon II in PGHF32 genes.

| **Species** | **Enzyme** | **No. Exon** | **No. Intron** | **Mini-exon** | **Mini-exon length (nt)** | **Mini-exon sequence (nt)** | **Translated sequence (aa)** |
|---|---|---|---|---|---|---|---|
| ***A. tauschii*** | Ata6SFT-like | 4 | 3 | Yes | 9 | GATCCCAAC | **DPN** |
| ***A. tauschii*** | Ata1FEH-like | 7 | 6 | Yes | 9 | GATCCATCT | **DPS** |
| ***A. tauschii*** | Ata1FFT-like | 4 | 3 | Yes | 9 | GATCCCAAC | **DPN** |
| ***A. tauschii*** | Ata1SST-like | 4 | 3 | Yes | 9 | GATCCCAAC | **DPN** |
| ***A. tauschii*** | AtaInv1-like | 3 | 2 | No | --- | --- | **---** |
| ***A. tauschii*** | AtaVinv1-like | 4 | 3 | Yes | 9 | GATCCCAAC | **DPN** |
| ***A. tauschii*** | AtaCwinv1-like | 7 | 6 | Yes | 9 | GATCCCAAC | **DPN** |
| ***H. vulgare*** | Hv1FFT | 4 | 3 | Yes | 9 | GATCCCAAC | **DPN** |
| ***H. vulgare*** | Hv6FEH | 6 | 5 | No | --- | --- | **---** |
| ***H. vulgare*** | HvInv1 | 4 | 3 | Yes | 9 | GATCCAAAC | **DPN** |
| ***H. vulgare*** | HvCwinv1-like | 7 | 6 | Yes | 9 | GATCCAAAT | **DPN** |
| ***H. vulgare*** | Hv1FEH | 7 | 6 | Yes | 9 | GATCCATCT | **DPS** |
| ***H. vulgare*** | HvInv2 | 4 | 3 | Yes | 9 | GATCCCAAC | **DPN** |
| ***H. vulgare*** | Hv1SST | 4 | 3 | Yes | 9 | GATCCCAAC | **DPN** |
| ***H. vulgare*** | Hv6SFT | 4 | 3 | Yes | 9 | GATCCCAAC | **DPN** |
| ***A. officinalis*** | Ao1SST | 8 | 7 | Yes | 8 | ATCCAAAT | **PN** |
| ***A. officinalis*** | Ao1FFT1 | 8 | 7 | Yes | 9 | GATCCTAGC | **DPS** |
| ***A. officinalis*** | Ao6GFFT | 8 | 7 | Yes | 9 | GATCCCAGC | **DPS** |
| ***A. officinalis*** | Ao6FEH | 6 | 5 | No | --- | --- | **---** |
| ***A. officinalis*** | AoCwinv1-like | 6 | 5 | No | --- | --- | **---** |
| ***A. officinalis*** | AoCwinv2-like | 7 | 6 | Yes | 9 | GATCCTAAT | **DPN** |
| ***O. sativa*** | OsInv1 | 5 | 4 | Yes | 9 | GATCCGAAT | **DPN** |

| | | | | | | | |
|---|---|---|---|---|---|---|---|
| ***O. sativa*** | OsInv2 | 7 | 6 | Yes | 9 | GATCCGAAC | **DPN** |
| ***O. sativa*** | OsInv3 | 3 | 2 | No | --- | --- | **---** |
| ***O. sativa*** | OsInv4 | 7 | 6 | Yes | 9 | GATCCGAAC | **DPN** |
| ***O. sativa*** | OsCwinv1 | 7 | 6 | Yes | 9 | GATCCGAAC | **DPN** |
| ***O. sativa*** | OsCwinv2 | 7 | 6 | Yes | 9 | GATCCGAAC | **DPN** |
| ***O. sativa*** | OsCwinv4 | 7 | 6 | Yes | 9 | GATCCGAAC | **DPN** |
| ***O. sativa*** | OsCwinv6 | 6 | 5 | Yes | 9 | GATCCGAAT | **DPN** |
| ***Z. mays*** | ZmInv1 | 7 | 6 | Yes | 9 | GATCCGAAC | **DPN** |
| ***Z. mays*** | ZmInv2 | 7 | 6 | Yes | 9 | GATCCGAAC | **DPN** |
| ***Z. mays*** | ZmCwinv1 | 7 | 6 | Yes | 9 | GACCCCAAC | **DPN** |
| ***Z. mays*** | ZmCwinv2 | 7 | 6 | Yes | 9 | GATCCCAAC | **DPN** |
| ***Z. mays*** | ZmCwinv3 | 6 | 5 | Yes | 9 | GATCCAAAT | **DPN** |
| ***Z. mays*** | ZmCwinv4 | 6 | 5 | Yes | 9 | GATCCGAAT | **DPN** |
| ***Z. mays*** | Zm6,1-FEH | 7 | 6 | Yes | 9 | GATCCCAAC | **DPN** |
| ***A. thaliana*** | AthVinv1 | 7 | 6 | Yes | 9 | GATCCTAAT | **DPN** |
| ***A. thaliana*** | AthVinv2 | 7 | 6 | Yes | 9 | GATCCTAAC | **DPN** |
| ***A. thaliana*** | AthCwinv1 | 7 | 6 | Yes | 9 | GATCCTAAT | **DPN** |
| ***A. thaliana*** | AthCwinv2 | 5 | 4 | Yes | 9 | GATCCGAAT | **DPN** |
| ***A. thaliana*** | AthCwinv4 | 6 | 5 | Yes | 9 | GATCCAAAT | **DPN** |
| ***A. thaliana*** | AthCwinv5 | 4 | 3 | No | --- | --- | **---** |
| ***A. thaliana*** | AthCwinv3 (6-FEH) | 7 | 6 | Yes | 9 | GATCCTAAT | **DPN** |
| ***A. thaliana*** | AthCwinv6 (6,1-FEH) | 7 | 6 | Yes | 9 | GATCCAAAC | **DPN** |
| ***B. vulgaris*** | Bv6FEH | 7 | 6 | Yes | 8 | AT<u>CCTAAT</u> | **PN** |
| ***B. vulgaris*** | BvVinv | 8 | 7 | Yes | 9 | GATCCAAAC | **DPN** |
| ***B. vulgaris*** | BvCwinv | 7 | 6 | Yes | 9 | GATCCTAAT | **DPN** |
| ***T. aestivum*** | Ta1FEHw2 | 7 | 6 | Yes | 9 | GATCCATCT | **DPS** |
| ***T. aestivum*** | Ta1FFTA | 4 | 3 | Yes | 9 | GATCCCAAC | **DPN** |

| | | | | | | | |
|---|---|---|---|---|---|---|---|
| ***T. aestivum*** | Ta1FFTB | 4 | 3 | Yes | 9 | GATCCCAAC | **DPN** |
| ***T. aestivum*** | Ta1SST | 4 | 3 | Yes | 9 | GATCCCAAC | **DPN** |
| ***T. aestivum*** | Ta6FEH | 6 | 5 | Yes | 9 | GATCCGAAC | **DPN** |
| ***T. aestivum*** | Ta6SFT | 4 | 3 | Yes | 9 | GATCCCAAC | **DPN** |
| ***T. aestivum*** | Ta6,1FEH | 9 | 8 | Yes | 9 | GATCCATGT | **DPC** |
| ***T. aestivum*** | Ta1FEHw1 | 7 | 6 | Yes | 9 | GATCCATCT | **DPS** |
| ***T. aestivum*** | Ta6KEHw1 | 9 | 8 | Yes | 9 | GATCCATGT | **DPC** |
| ***T. aestivum*** | Ta6KEHw2 | 9 | 8 | Yes | 9 | GATCCATGT | **DPC** |
| ***T. aestivum*** | Ta1FEHw3 | 7 | 6 | Yes | 9 | GATCCATCT | **DPS** |
| ***T. aestivum*** | TaInv1-like | 3 | 2 | No | --- | --- | **---** |
| ***T. aestivum*** | TaVinv2-like | 4 | 3 | Yes | 9 | GATCCCAAC | **DPN** |
| ***T. aestivum*** | TaVinv3-like | 4 | 3 | Yes | 9 | GATCCCAAC | **DPN** |
| ***T. aestivum*** | TaCwinv1-like | 7 | 6 | Yes | 9 | GATCCCAAC | **DPN** |
| ***T. aestivum*** | TaCwinv2-like | 7 | 6 | Yes | 9 | GATCCCAAC | **DPN** |
| ***T. aestivum*** | TaCwinv3-like | 7 | 6 | Yes | 9 | GATCCCAAC | **DPN** |
| ***D. carota*** | DcCwinv1-like | 7 | 6 | Yes | 9 | GATCCTAAT | **DPN** |
| ***D. carota*** | DcCwinv2-like | 7 | 6 | Yes | 9 | GATCCAAAT | **DPN** |
| ***D. carota*** | DcCwinv3-like | 7 | 6 | Yes | 9 | GATCCCAAT | **DPN** |
| ***D. carota*** | DcVinv1-like | 8 | 7 | Yes | 9 | GATCCTAAT | **DPN** |
| ***D. carota*** | DcVinv2-like | 7 | 6 | Yes | 9 | GATCCTAAC | **DPN** |
| ***D. carota*** | DcVinv3-like | 7 | 6 | Yes | 9 | GATCCTAAT | **DPN** |
| ***A. tequilana*** | Atq1SST-1 | 8 | 7 | Yes | 9 | GATCCCAAT | **DPN** |
| ***A. tequilana*** | Atq1SST-2 | 8 | 7 | Yes | 9 | GATCCTAAT | **DPN** |
| ***A. tequilana*** | Atq1SST-3 | 8 | 7 | Yes | 9 | GATCCCAAT | **DPN** |
| ***A. tequilana*** | Atq6GFFT-1 | 8 | 7 | Yes | 9 | GATCCATGT | **DPC** |
| ***A. tequilana*** | Atq6GFFT-2 | 8 | 7 | Yes | 9 | GATCCAAGT | **DPS** |
| ***A. tequilana*** | Atq1FFT | 8 | 7 | Yes | 9 | GATCCCAGC | **DPS** |

| | | | | | | | |
|---|---|---|---|---|---|---|---|
| ***A. tequilana*** | AtqVinv1 | 6 | 5 | Yes | 9 | GATCCCAAC | **DPN** |
| ***A. tequilana*** | AtqVinv2 | 8 | 7 | Yes | 9 | GATCCCAAT | **DPN** |
| ***A. tequilana*** | AtqVinv3-like | 8 | 7 | Yes | 9 | GATCCCAAC | **DPN** |
| ***A. tequilana*** | AtqVinv4-like | 8 | 7 | Yes | 9 | GATCCCAAT | **DPN** |
| ***A. tequilana*** | AtqVinv5-like | 8 | 7 | Yes | 9 | GATCCCAAC | **DPN** |
| ***A. tequilana*** | AtqInv1 | 7 | 6 | Yes | 9 | GATCCTGAC | **DPD** |
| ***A. tequilana*** | AtqInv2 | 6 | 5 | Yes | 8 | ATCCTAAT | **PN** |
| ***A. tequilana*** | AtqCwinv-1 | 7 | 6 | Yes | 9 | GATCCAAAT | **DPN** |
| ***A. tequilana*** | AtqCwinv-2 | 7 | 6 | Yes | 9 | GATCCTAAT | **DPN** |
| ***A. tequilana*** | AtqFEH-1 | 6 | 5 | No | --- | --- | **---** |
| ***A. tequilana*** | AtqFEH-2 | 6 | 5 | No | --- | --- | **---** |
| ***A. tequilana*** | AtqFEH-3 | 5 | 4 | No | --- | --- | **---** |
| ***A. tequilana*** | AtqFEH-4 | 6 | 5 | No | --- | --- | **---** |
