# Supplementary figures and images for: Evidence for Light and Tissue Specific Regulation of Genes Involved in Fructan Metabolism in Agave tequilana
Source: Plants (Basel). 2022 Aug 19;11(16):2153. doi: 10.3390/plants11162153 (PMC9412663; doi:10.3390/plants11162153)

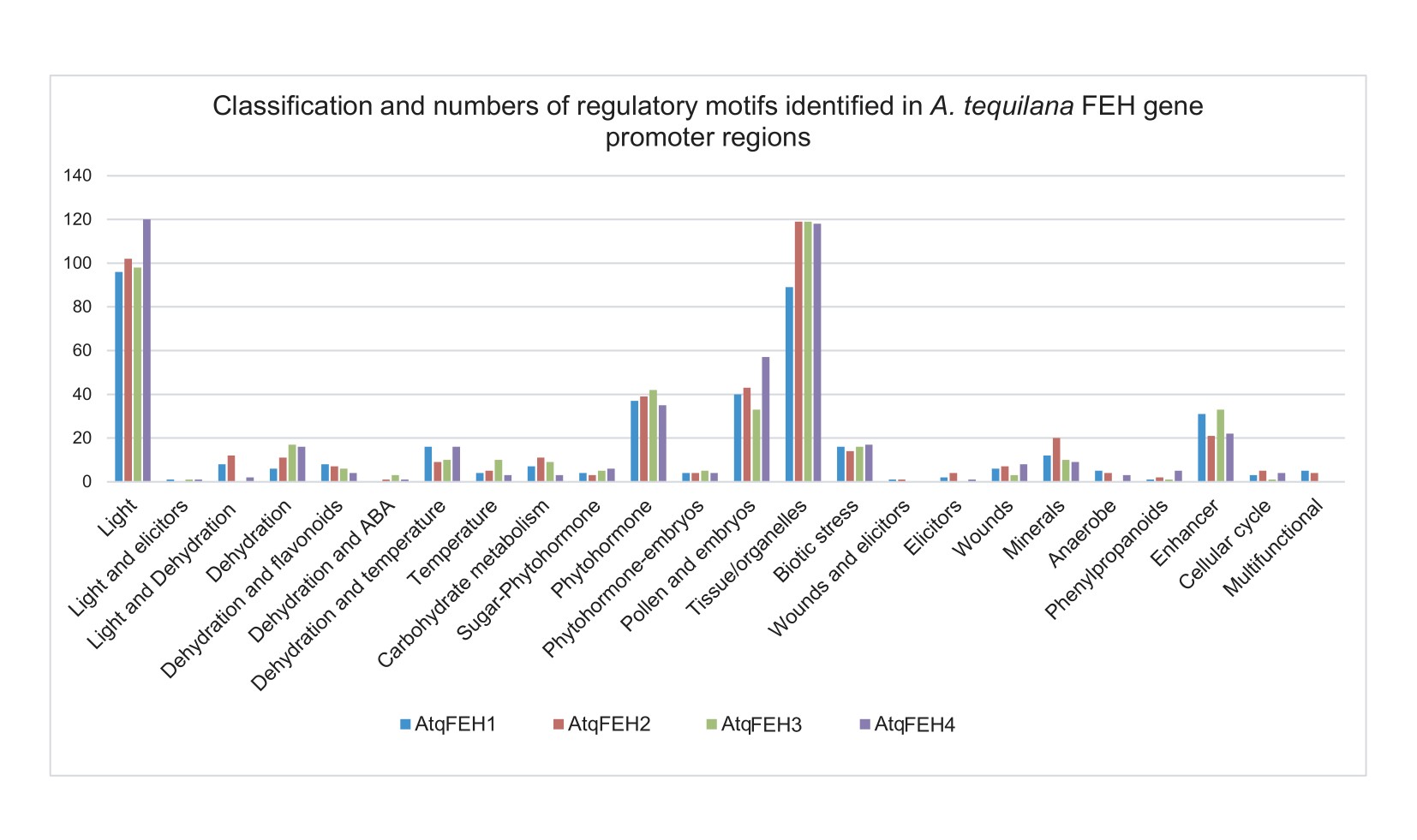

Supplement: Supplementary file 1 [file plants-11-02153-s001.zip › Supplementary figure S2.jpg]

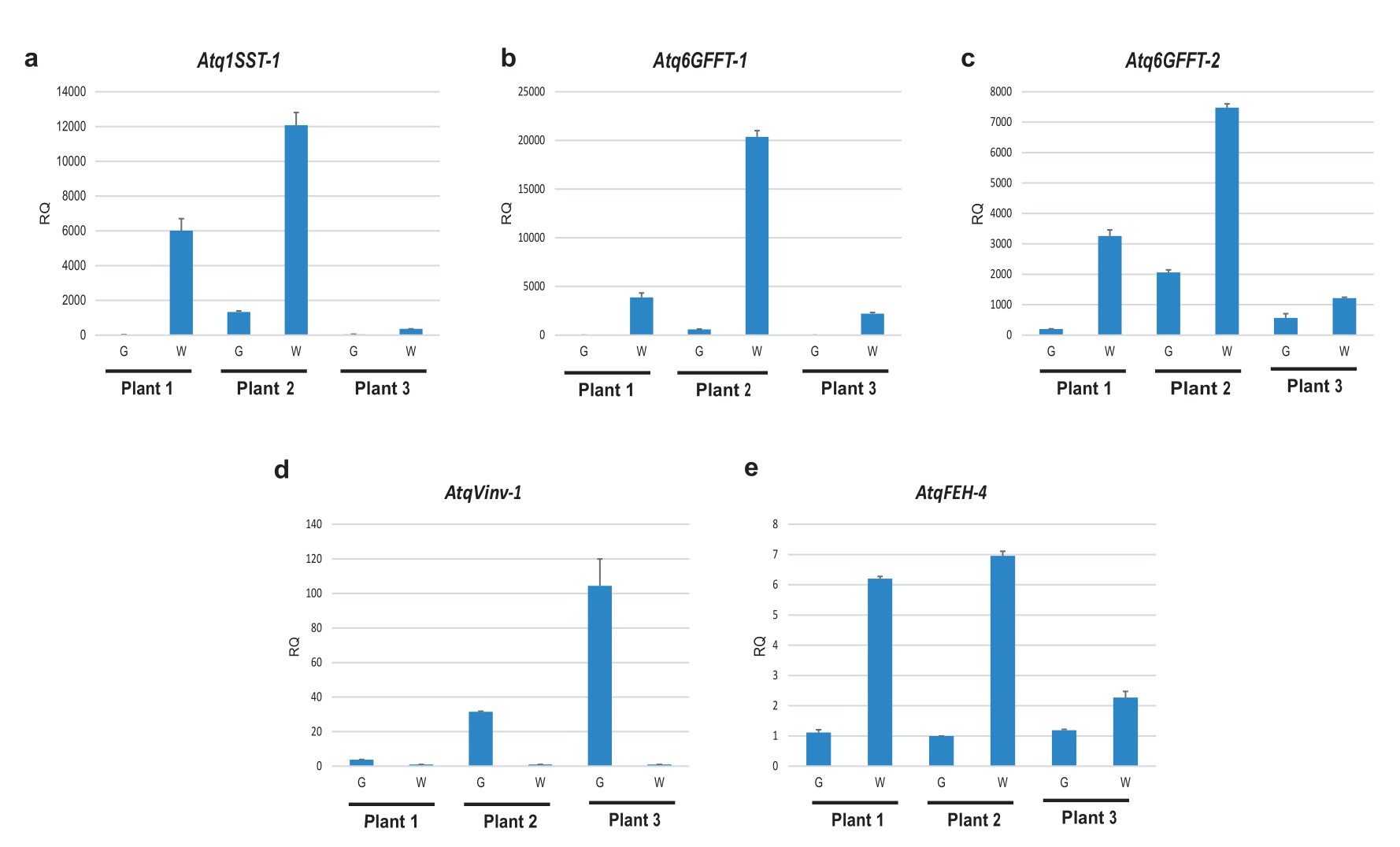

Supplement: Supplementary file 1 [file plants-11-02153-s001.zip › Supplementary figure S3.jpg]
